# Phase 1 dose-finding and pharmacokinetic study of eribulin-liposomal formulation in patients with solid tumours

**DOI:** 10.1038/s41416-019-0377-x

**Published:** 2019-01-25

**Authors:** T. R. Jeffry Evans, Emma Dean, L. Rhoda Molife, Juanita Lopez, Malcolm Ranson, Fatima El-Khouly, Ishtiaq Zubairi, Claudio Savulsky, Larisa Reyderman, Yan Jia, Lorna Sweeting, Alastair Greystoke, Jorge Barriuso, Rebecca Kristeleit

**Affiliations:** 1 Beatson West of Scotland Cancer Centre, Glasgow, UK; 2 University of Glasgow, Glasgow, UK; 3 The Christie NHS Foundation Trust, Manchester, UK; 4 The Royal Marsden Hospital NHS Foundation Trust and The Institute of Cancer Research, London, UK; 5 The Christie NHS Foundation Trust, Manchester, UK; 6 UCL Cancer Institute, London, UK; 7 Eisai Ltd., Hatfield, UK; 8 Eisai Inc., Woodcliff Lake, NJ USA; 9 Northern Institute for Cancer Research, Newcastle University, Newcastle upon Tyne, UK; 10 UCL Cancer Institute, London, UK

**Keywords:** Breast cancer

## Abstract

**Background:**

This phase 1 study examined the safety, tolerability, pharmacokinetics and preliminary efficacy of eribulin-liposomal formulation (eribulin-LF) in patients with advanced solid tumours.

**Methods:**

Eligible patients with ECOG PS 0–1 were treated with eribulin-LF either on day 1 every 21 days (Schedule 1), or on days 1 and 15 every 28 days (Schedule 2). Doses ranged from 1.0 to 3.5 mg/m^2^, with dose escalation in a 3 + 3 design. The dose-expansion phase evaluated eribulin-LF in select tumour types. Primary objectives: maximum tolerated dose (MTD) and the recommended dose/schedule of eribulin-LF.

**Results:**

Totally, 58 patients were enroled (median age = 62 years). The MTD was 1.4 mg/m^2^ (Schedule 1) or 1.5 mg/m^2^ (Schedule 2), the latter dose selected for the dose-expansion phase. Dose-limiting toxicity (DLTs) in Schedule 1: hypophosphatemia and increased transaminase levels. DLTs in Schedule 2: stomatitis, increased alanine aminotransferase, neutropenia and febrile neutropenia. The pharmacokinetic profile of eribulin-LF showed a similar half-life to that of eribulin (~30 h), but with a 5-fold greater maximum serum concentration and a 40-fold greater area-under-the-curve. Eribulin-LF demonstrated clinical activity with approximately 10% of patients in both schedules achieving partial responses.

**Conclusions:**

Eribulin-LF was well tolerated with a favourable pharmacokinetic profile. Preliminary evidence of clinical activity in solid tumours was observed.

## Background

Eribulin-liposomal formulation (eribulin-LF) is a new formulation designed to improve the therapeutic index of the drug by encapsulating eribulin mesylate in the interior of the water phase of liposomes. Eribulin mesylate is a nontaxane antineoplastic agent that inhibits microtubule dynamics and prevents tumour cell proliferation.^1–3^ Eribulin is a synthetic analogue of the natural product, halichondrin B, isolated from the marine sponge *Halichondria okadai*.^3^ Eribulin has a novel mode of action that distinguishes it from other tubulin-targeted drugs, including vinca alkaloids: it leads to irreversible mitotic blockade by binding to a small number of high-affinity sites on growing (plus) ends of microtubules to inhibit their growth, without having a measurable effect on microtubule shortening.^1,2,4^ Nonmitotic effects of eribulin include suppressing cancer cell migration and invasion, reversing the epithelial-to-mesenchymal transition that has been associated with a malignant phenotype and tumour vascular remodelling resulting in increased perfusion.^4,5^

Eribulin is approved as a monotherapy for patients with metastatic breast cancer (MBC) who have previously been treated with at least 2 (in the United States) or 1 (in Europe) prior chemotherapeutic regimens, including an anthracycline and a taxane, in either adjuvant or metastatic settings.^6–8^ Furthermore, in a phase 3 clinical trial of patients with advanced liposarcoma or leiomyosarcoma, eribulin significantly prolonged overall survival compared with dacarbazine, and consequently, eribulin is also approved for unresectable or metastatic liposarcoma in patients who have already received an anthracycline-containing regimen.^7–9^ A pooled analysis of 2 randomised phase 3 studies showed that eribulin could significantly improve overall survival in patients with human epidermal growth factor receptor 2 (HER2)-negative breast cancer and in patients with triple-negative disease.^10^

The potential efficacy of eribulin can be compromised by dose reductions and omissions due to toxicities, the most common being neutropenia and peripheral neuropathy.^7^ However, the use of PEGylated liposomes to encapsulate drugs can help reduce systemic toxicity^11^ and optimise exposure to drug. The liposome platform facilitates the selective delivery of pharmacologic agents to tumour cells, increasing selective uptake by these cells and, therefore, control of drug delivery.^12^ PEGylated liposomal drugs offer several additional advantages, including enhanced permeability and longer retention in tumour tissues and improved efficacy.^11,13^

Preclinical experiments of eribulin-LF in mice suggested improved pharmacokinetics characterised by slower clearance, a lower volume of distribution, and a 1.5-fold higher exposure to free eribulin compared with eribulin mesylate.^14,15^ Eribulin-LF has also demonstrated improved antitumour activity in a human pharynx squamous cell carcinoma xenograft model compared with eribulin mesylate.^14^

## Objectives

This phase 1 first-in-human trial aimed to identify the maximum tolerated dose (MTD), recommended phase 2 dose and schedule, and to assess the safety, tolerability, pharmacokinetic and preliminary efficacy of eribulin-LF in patients with advanced or metastatic solid tumours, including breast, ovarian or endometrial cancers.

## Methods

### Study design

This phase 1, open-label, multicenter study comprised a dose-escalation phase and a dose-expansion phase. The dose-escalation phase consisted of a 3 + 3 design with 2 schedules: In Schedule 1, eribulin-LF was administered as a 60-min intravenous (IV) infusion on day 1 of a 21-day cycle and in Schedule 2, eribulin-LF was administered as a 60-min IV infusion on day 1 and day 15 of a 28-day cycle. These schedules were chosen in anticipation of a longer elimination half-life with eribulin-LF compared with eribulin, which is administered on day 1 and 8 of a 28-day cycle. The MTD was defined as the highest dose level at which ≤1 of 6 patients experienced a dose-limiting toxicity (DLT).

The dose-expansion phase confirmed safety and tolerability of the MTD defined in the dose-escalation part of the study and preliminarily assessed clinical activity of eribulin-LF in patients with endometrial, ovarian or HER2-negative breast cancer. Study treatment continued until disease progression, unacceptable toxicity or withdrawal of patient consent.

A clinical dose of eribulin mesylate 1.4 mg/m^2^ is equivalent to 1.23 mg/m^2^ of eribulin expressed as free base. Therefore, eribulin administered on day 1 and day 8 of a 21-day cycle is equivalent to 2.46 mg/m^2^ total dose per cycle. The starting dose selected for this study was 1.0 mg/m^2^, which represents a 60% reduction in the approved eribulin dose administered per cycle (2.46 mg/m^2^). Dose increments of 0.5 mg/m^2^ were planned to reach 3.5 mg/m^2^.

#### Inclusion and exclusion criteria

Patients aged ≥ 18 years with histologically or cytologically proven advanced solid tumours refractory to standard therapies were enroled. In the dose-expansion phase, the following tumours were permitted: endometrial cancer after failure of prior chemotherapy for recurrent, metastatic or high-risk disease; ovarian cancer after failure of prior chemotherapy or intolerance of platinum agents (including patients with platinum-sensitive and platinum-refractory disease); and breast cancer (HER2-negative subtype) after failure of previous standard (anthracycline and taxane) chemotherapy treatments. Patients were required to have had ≥1 measurable lesion based on Response Evaluation Criteria In Solid Tumours (RECIST) version (v) 1.1 (dose-expansion phase), an Eastern Cooperative Oncology Group performance status ≤ 1, and adequate liver, renal and bone marrow function.

Key exclusion criteria included patients having had any anticancer therapy within 21 days prior to study entry for cytotoxic agents and targeted agents, or within 30 days for an investigational agent, patients with pre-existing peripheral neuropathy of National Cancer Institute Common Terminology Criteria for Adverse Events (CTCAE) grade > 1, patients who had previously been treated with eribulin, and patients who had not recovered (to grade < 2) from acute toxicities associated with prior anticancer therapy or had received radiation therapy encompassing > 30% of the bone marrow.

#### Dose escalation and reduction

Dose escalation of eribulin-LF was based on safety and tolerability during the first treatment cycle. Severe toxicities (defined and graded as CTCAE version 4.03, grade ≥ 3) during later treatment cycles were also considered. DLTs were defined as any of the following: neutropenia grade 4 lasting > 5 days; neutropenia grade 3 or 4 complicated by fever and/or infection (absolute neutrophil count [ANC] < 1.0 × 10^9^/L, fever ≥ 38.5 °C); grade 4 thrombocytopenia of any duration; grade 3 thrombocytopenia complicated by bleeding and/or requiring platelet or blood transfusion; grade 3 or 4 hypersensitivity reactions, symptomatic bronchospasm requiring parenteral medication(s) with or without urticaria or allergy-related oedema/angioedema and other grade 3/4 clinically significant nonhematologic toxicities (except for inadequately treated nausea and/or vomiting) considered related to the study drug, including peripheral neuropathy. During the dose-expansion phase, pre-specified dose interruptions and dose reductions based on prior data from eribulin studies were permitted if eribulin-LF-related toxicity occurred (see Supplementary Table 1).

#### Safety analyses

Safety assessments included monitoring and recording all adverse events (AEs) and serious AEs (SAEs). Patients underwent monitoring of haematology, blood chemistry, urine values, vital signs, electrocardiograms (at screening, baseline, day 1 and day 15 of each cycle and within 30 days after the last dose of study treatment) and physical examinations (at screening, baseline, day 1 and day 15 of cycle 1, day 1 of each subsequent treatment cycle and within 30 days after the last dose of study treatment). Hypersensitivity reactions were considered events of special interest.

#### Pharmacokinetic analyses

Blood samples for pharmacokinetic analyses were collected during cycle 1 and cycle 3 in the dose-escalation phase and cycle 1 in the dose-expansion phase. For patients receiving eribulin-LF in Schedule 1, blood samples were collected on day 1 at predose, 15 min after the start of infusion, 5 min after the end of infusion, 0.5, 1, 2, 4, 6, 8 and 24 h postdose, and on days 4, 7, 9 and 11. For patients receiving eribulin-LF in Schedule 2, blood samples were collected on day 1 and day 15 at predose, 15 min after the start of infusion, 5 min after the end of infusion, 0.5, 1, 2, 4, 6, 8 and 24 h postdose, and on days 4, 7, 9 and 11.

Urine samples for pharmacokinetic analyses were collected in the dose-escalation cohort during cycle 1 and cycle 3. For patients receiving eribulin-LF according to Schedule 1, urine samples were collected predose, and at 0−24 h (day 1), 24−48 h (day 2), 48−72 h (day 3) and 72−96 h (day 4). For patients receiving eribulin-LF according to Schedule 2, urine samples were collected predose, and at 0−24 h (day 1/day 15), 24−48 h (day 2/day 16), 48−72 h (day 3/day 17) and 72−96 h (day 4/day 18).

Eribulin plasma concentrations were quantified by liquid chromatography with tandem mass spectrometry methodology using a previously validated assay.^16^ Eribulin concentrations refer to total eribulin.

#### Efficacy analyses

Tumours were imaged using computed tomography or magnetic resonance imaging, and assessed according to RECIST v1.1^17^ by study site investigators. In accordance with RECIST v1.1 recommendations, a confirmatory scan was not required, as this was a phase 1 study where safety is the primary endpoint. Imaging was performed at screening (pretreatment) and after every other cycle (i.e., every 6 weeks after cycle 1, day 1 for Schedule 1 and every 8 weeks after cycle 1, day 1 for Schedule 2). Patients who discontinued study treatment during the dose-escalation phase without disease progression continued to undergo tumour assessments every 9 weeks (for Schedule 1) or every 8 weeks (Schedule 2). Patients in the dose-expansion phase who discontinued study treatment without disease progression had tumour assessments every 8 weeks. Disease-control rate is defined as the proportion of patients who achieved a best overall response of complete response or partial response or stable disease (SD). In order for SD to be deemed the best overall response, it must occur ≥ 5 weeks (or ≥7 weeks for Schedule 2) following the first dose of study drug.

## Statistical analysis

Safety and efficacy analyses were based on the safety analysis set, which included all patients who received ≥1 dose of study drug and had ≥1 postdose safety assessment. Pharmacokinetic analyses were based on the pharmacokinetic analysis set, which included all patients with sufficient pharmacokinetic data to derive ≥1 parameter. The dose-finding analysis set included all patients in the dose-escalation phase of the study who completed cycle 1 treatment and were evaluable for DLTs, as well as those who discontinued during cycle 1 due to DLTs. Actual dose intensity is defined as: total dose received over all treatment cycles (mg/m^2^)/(duration of treatments in days/7).

Descriptive summary statistics (mean, standard deviation (SD), median, minimum and maximum) were determined for all continuous data (e.g., clinical laboratory test results, vital signs measurements and changes from baseline), and the number and percentage of patients were determined for all categorical data (e.g., baseline patient demographics and clinical characteristics). The number and percentage of patients reporting treatment emergent adverse events (TEAEs) were summarised by both CTCAE grade and relationship to study drug. Efficacy parameters were summarised separately for patients in the expanded cohorts for each tumour type. Eribulin pharmacokinetic parameters were derived from plasma concentrations by noncompartmental analysis using actual times. Statistical analyses were performed using SAS, v 9.3 (Cary, NC).

## Results

### Patient disposition and demographics

The study was conducted between 11 December 2012 and 16 May 2016 at four sites in the United Kingdom. A total of 71 patients were screened, 58 of whom received treatment (Supplementary Figure 1): 20 patients in Schedule 1 and 38 patients in Schedule 2. Baseline demographics are shown in Table 1. Among 20 patients who received eribulin-LF according to Schedule 1, the majority were male (11 patients; 55.0%) and the median age was 61.0 years. The most common cancer types were pleural mesothelioma and uterine cervix cancer (15.0% each). Among the 38 patients who received eribulin-LF according to Schedule 2, most were female (29 patients; 76.3%), the median age was 62.5 years, and the most common cancer types were sites of primary lesion were ovarian (26.3%), breast (23.7%), and colorectal cancers (23.7%). Schedule 1 included patients with more diverse tumour types, whereas Schedule 2 included patients with specific tumour types (endometrial, ovarian and breast cancers) for the expansion part of the study. Overall, the majority of patients (>65%) had either colorectal (*n* = 11), breast (*n* = 10), ovarian (*n* = 10) or endometrial (*n* = 7) cancers.

**Table 1:** Patient demographics and disease characteristics

Parameter	Schedule 1 (*n* = 20)	Schedule 2 (*n* = 38)	All patients (*n* = 58)
Age, years			
Median (range)	61.0 (33−75)	62.5 (40−70)	62.0 (33−75)
Sex, *n* (%)			
Male	11 (55.0)	9 (23.7)	20 (34.5)
Female	9 (45.0)	29 (76.3)	38 (65.5)
Race, *n* (%)			
White	19 (95.0)	36 (94.7)	55 (94.8)
Black or African American	1 (5.0)	1 (2.6)	2 (3.4)
Other	0	1 (2.6)	1 (1.7)
ECOG PS, *n* (%)			
0	4 (20.0)	16 (42.1)	20 (34.5)
1	16 (80.0)	22 (57.9)	38 (65.5)
Prior systemic therapies, *n* (%)			
1	6 (30.0)	4 (10.5)	10 (17.2)
2	9 (45.0)	7 (18.4)	16 (27.6)
3	3 (15.0)	12 (31.6)	15 (25.9)
4	1 (5.0)	4 (10.5)	5 (8.6)
5−14	1 (5.0)	11 (28.9)	12 (20.7)
Tumour type, *n* (%)			
Breast	1 (5.0)	9 (23.7)	10 (17.2)
Cholangiocarcinoma	2 (10.0)	0	2 (3.4)
Colorectal^a^	2 (10.0)	9 (23.7)	11 (19.0)
Endometrial	1 (5.0)	6 (15.8)	7 (12.1)
Gall bladder	2 (10.0)	0	2 (3.4)
NSCLC	1 (5.0)	2 (5.3)	3 (5.2)
Ovarian	0	10 (26.3)	10 (17.2)
Pancreatic	2 (10.0)	1 (2.6)	3 (5.2)
Pleural mesothelioma	3 (15.0)	0	3 (5.2)
Uterine cervix	3 (15.0)	0	3 (5.2)
Other^b^	3 (15.0)	1 (2.6)	4 (6.9)

^a^Includes colon, rectum and large intestine (excludes appendix)

^b^Includes adrenal glands, duodenal, oesophageal and laryngeal cancers.

*ECOG PS* Eastern Cooperative Oncology Group performance score, *NSCLC* non–small-cell lung cancer

#### Maximum tolerated dose

DLTs in the dose-escalation phase are summarised in Table 2. For Schedule 1, the MTD for eribulin-LF was determined to be 1.4 mg/m^2^ on day 1 every 21 days. At the 1.5 mg/m^2^ dose in Schedule 1, 2 patients had DLTs (grade 4 hypophosphatemia [*n* = 1], grade 4 increased alanine aminotransferase/aspartate aminotransferase levels [ALT/AST; *n* = 1]). An additional 3 patients were evaluated at a dose of 1.0 mg/m^2^, none of whom experienced a DLT. Therefore, an intermediate dose of 1.4 mg/m^2^ eribulin-LF in Schedule 1 was evaluated in 6 patients, with none experiencing a DLT. For Schedule 2, the MTD was determined to be 1.5 mg/m^2^ on day 1 and day 15 of a 28-day cycle, with 2 DLTs reported at that dose in 1 patient (grade 4 febrile neutropenia and grade 3 stomatitis). At the 2 mg/m^2^ dose, 3 patients were treated and 2 had DLTs (grade 3 increased ALT [*n* = 1] and grade 4 neutropenia [*n* = 1]).

**Table 2:** Dose-limiting toxicities among patients^a^ in the dose-escalation phase

Schedule, Patient, *n*	Eribulin-LF dose	Patient, *n*	DLT
1 (18)	1.5 mg/m^2^	1	Grade 4 hypophosphatemia
		1	Grade 4 increased ALT/AST
2 (12)	1.5 mg/m^2^	1	Grade 4 febrile neutropenia
Grade 3 stomatitis			
	2.0 mg/m^2^	1	Grade 3 increased ALT
		1	Grade 4 neutropenia

^a^Determination of the MTD was based on the dose-finding analysis set, which included all patients in the dose-escalation part who completed cycle 1 treatment and were evaluated for DLTs, and those who discontinued cycle 1 during the DLT. Of the 35 patients treated in the dose-escalation phase, 30 were evaluable for DLTs (*n* = 18 in Schedule 1 and *n* = 12 in Schedule 2)

*ALT* alanine aminotransferase, *DLT* dose-limiting toxicity, *LF* liposomal formulation, *MTD* maximum tolerated dose

#### Exposure

The median (range) number of cycles was 3 (1–26) for Schedule 1 and 2 (1–12) for Schedule 2. Of the 58 patients who received treatment, 43 (74.1%) discontinued due to objective disease progression, 9 (15.5%) due to clinical disease progression, 3 (5.2%) due to patient choice, 1 (1.7%) due to toxicity, 1 (1.7%) due to AEs, and 1 (1.7%) due to death >30 days after the last dose of study treatment. The duration of treatment is summarised in Supplementary Figure 2; the median durations of treatment were similar for Schedule 1 and Schedule 2 (9 and 8 weeks, respectively).

Due to dosing frequency, the dose intensity differed between Schedules 1 and 2 at their respective MTDs. The planned dose intensity in Schedule 2 was 60% higher than in Schedule 1 (0.75 vs. 0.47 mg/m^2^/week). At the MTD dose of 1.4 mg/m^2^ for Schedule 1, the mean actual dose intensity (defined as total dose [mg/m^2^] received/[duration of treatment in days/7]) was 0.5 mg/m^2^/week per patient, with a mean relative dose intensity (defined as actual dose intensity/planned dose intensity) of 1.0 (SD = 0.05). For the Schedule 2 MTD of 1.5 mg/m^2^ (including both dose-escalation and dose-expansion patients, *n* = 32), the actual dose intensity was 0.6 mg/m^2^/week per patient, with a mean relative dose intensity of 0.7 (SD = 0.19).

More patients in Schedule 2 had TEAEs leading to dose interruption than patients in Schedule 1 (60.5% vs. 25.0%). There was a similar rate of dose reduction due to TEAEs in Schedules 1 and 2 (5.0% vs. 7.9%). Overall, 5 patients (8.6%) experienced TEAEs that led to study-drug withdrawal (Supplementary Table 2). The incidence of treatment-related TEAEs leading to study-drug dose reduction was similar between Schedules 1 and 2 (5.0% vs. 5.3%); the most common reasons were nonhematological toxicities (ALT level increase/hypophosphatemia). Treatment-related TEAEs leading to study-drug dose interruption were more frequent with Schedule 2 than with Schedule 1 (50% vs. 20%), with neutropenia being the most frequent cause of drug interruption in Schedule 2 (14 patients; 36.8%).

#### Safety

Overall, 89.7% of TEAEs were considered treatment related; this was reported in 18 (90%) patients receiving Schedule 1 and 34 (89.5%) patients receiving Schedule 2 (Supplementary Table 2). Overall, SAEs were reported in 41.4% of patients and TEAEs of grade ≥ 3 were reported in 48.2% of patients (60% and 42.1% for Schedules 1 and 2, respectively).

The most frequent TEAEs with Schedule 1 were alopecia (50.0%), decreased appetite (50.0%), constipation (40.0%), and diarrhoea (40.0%); and the most frequent TEAEs with Schedule 2 were neutropenia (42.1%), nausea (42.1%), fatigue (34.2%) and alopecia (34.2%). Overall, the most common grade 3/4 TEAEs were neutropenia (17.2%), hypophosphatemia (6.9%) and musculoskeletal chest pain (5.2%) (Table 3). More grade 3/4 events occurred in Schedule 1 compared with Schedule 2 (60.0% vs. 42.1%, respectively). However, the overall incidence of grade 3/4 neutropenia was higher in Schedule 2 compared with Schedule 1 (21.1% vs. 10.0%, respectively). A total of 3 (5.2%) patients experienced grade 3 hypophosphatemia, and 1 (1.7%) patient had grade 4 hypophosphatemia.

**Table 3:** TEAEs^a^ of grade 3 or 4 occurring in ≥2% of patients in any treatment group (all cycles)

TEAE, *n* (%)	Schedule 1 (*n* = 20)		Schedule 2 (*n* = 38)		All patients (*n* = 58)	
	Grade 3	Grade 4	Grade 3	Grade 4	Grade 3	Grade 4
Any	9 (45.0)	3 (15.0)	9 (23.7)	7 (18.4)	18 (31.0)	10 (17.2)
Blood and lymphatic system disorders						
Febrile neutropenia	0	1 (5.0)	1 (2.6)	1 (2.6)	1 (1.7)	2 (3.4)
Neutropenia	2 (10.0)	0	3 (7.9)	5 (13.2)	5 (8.6)	5 (8.6)
Gastrointestinal disorders						
Ascites	1 (5.0)	0	0	0	1 (1.7)	0
Nausea	0 (0)	0 (0)	1 (2.6)	0	1 (1.7)	0
Stomatitis	0 (0)	0 (0)	2 (5.3)	0	2 (3.4)	0
General disorders and administration-site conditions						
Device occlusion	1 (5.0)	0	0	0	1 (1.7)	0
Pyrexia	2 (10)	0 (0)	0	0	2 (3.4)	0
Infections and infestations						
Neutropenic sepsis	1 (5.0)	0	1 (2.6)	0	2 (3.4)	0
Sepsis	0	0	0	1 (2.6)	0	1 (1.7)
Upper respiratory tract infection	0	0	2 (5.3)	0	2 (3.4)	0
Urinary tract infection	0	0	2 (5.3)	0	2 (3.4)	0
Wound infection	0	0	1 (2.6)	0	1 (1.7)	0
Metabolism and nutrition disorders						
Hypernatremia	0	0	1 (2.6)	0	1 (1.7)	0
Hypomagnesemia	0	0	1 (2.6)	0	1 (1.7)	0
Hypophosphatemia	0	1 (5.0)	3 (7.9)	0	3 (5.2)	1 (1.7)
Musculoskeletal and connective tissue disorders						
Musculoskeletal chest pain	2 (10.0)	0 (0)	1 (2.6)	0	3 (5.2)	0
Investigations						
Alanine aminotransferase level increased	0	0	1 (2.6)	0	1 (1.7)	0
Respiratory, thoracic, and mediastinal disorders						
Pleuritic pain	1 (5.0)	0	0	0	1 (1.7)	0
Pulmonary embolism	1 (5.0)	0	0	0	1 (1.7)	0

^a^If a patient had two or more adverse events with the same preferred term with different CTCAE grades, the event with the highest grade was used.

*CTCAE* Common Terminology Criteria for Adverse Events, *TEAE* treatment-emergent adverse event

No deaths were reported during the study or within 30 days of the last dose of study drug. The most frequently reported SAEs were pyrexia (7 patients; 12.1%) and neutropenia (4 patients; 6.9%). Six patients each treated in Schedule 1 (30%) and in Schedule 2 (15.8%) had a treatment-related SAE. The most common treatment-related SAEs were neutropenia (6.9%), febrile neutropenia/neutropenic sepsis (5 individual patients; 8.6%) and pyrexia (3.4%) (Supplementary Table 3).

Overall, AEs of special interest were reported in 84.5% of patients, the most common being asthenia/fatigue (33 patients; 56.9%), alopecia (23 patients; 39.7%), neutropenia (21 patients; 36.2%) and peripheral neuropathy (narrow and broad terms: 11 patients; 19.0%; peripheral sensory neuropathy: 6 patients; 10.3%). The frequency of treatment-emergent hypersensitivity reactions was 10.0% (2 patients, both grade 2) in Schedule 1 vs. 7.9% (3 patients: 1 with grade 1 and 2 with grade 2) in Schedule 2. No grade ≥ 3 hypersensitivity reactions occurred.

#### Pharmacokinetic analyses

**Fig. 1:**
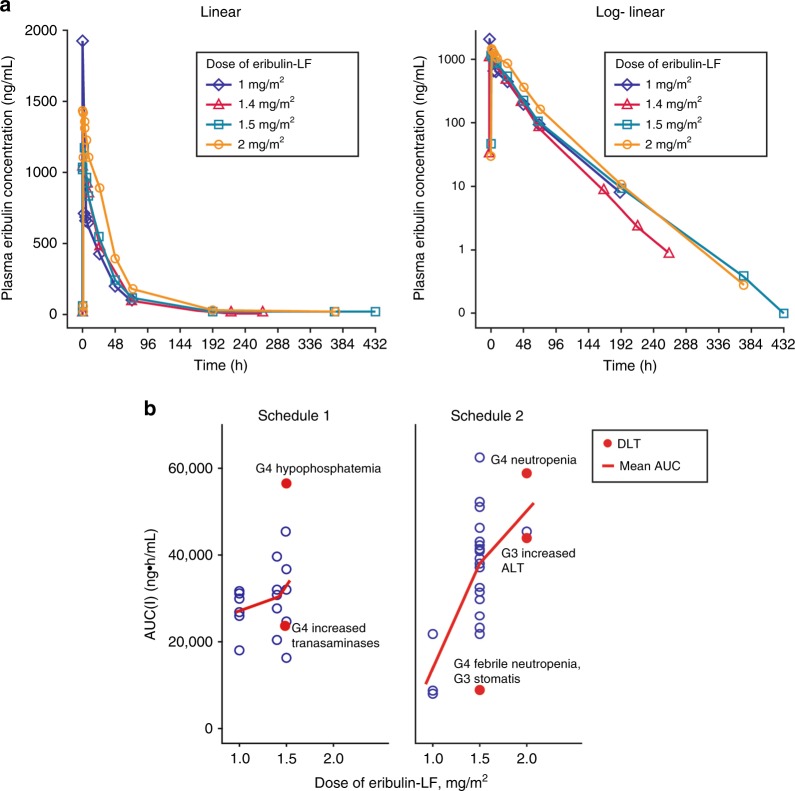
Pharmacokinetic profile of eribulin-LF. Mean plasma concentration–time curves of eribulin by dose of eribulin-LF on a linear scale and log-linear scale (**a**) and individual AUC versus eribulin-LF dose in Schedule 1 and Schedule 2 (**b**)—patients with DLTs are highlighted in red. *AUC(I)* area under the curve (infinity), *DLT* dose-limiting toxicity, *LF* liposomal formulation

Eribulin plasma-concentration profiles followed single-phase elimination (Fig. 1a). Exposure to eribulin-LF was dose dependent and independent of schedule (Figure 1a; Table 4). Eribulin-LF was eliminated with a mean half-life (*t*_1/2_) of approximately 30 h, with eribulin plasma concentrations quantifiable for up to 18 days postdose. Eribulin did not accumulate with multiple dosing. Eribulin exposure on cycle 3, day 1 was comparable with that following a single dose on cycle 1, day 1. Exposure to eribulin-LF was variable and overlapped across doses and schedules. There was no observed correlation between eribulin exposure (AUC) and the occurrence of DLTs (Fig. 1b). For example, grade 4 febrile neutropenia and grade 4 increase in transaminase levels in patients dosed with 1.5 mg/m^2^ eribulin-LF were associated with the lower eribulin exposure observed.

**Table 4:** Eribulin plasma pharmacokinetic parameters (dose-escalation phase; cycle 1, day 1)

Parameter, mean (standard deviation)	Schedule 1			Schedule 2		
	Eribulin-LF dose			Eribulin-LF dose		
	1.0 mg/m^2^ (*n* = 6)	1.4 mg/m^2^ (*n* = 6)	1.5 mg/m^2^ (*n* = 7)	1.0 mg/m^2^ (*n* = 3)	1.5 mg/m^2^ (*n* = 9)	2.0 mg/m^2^ (*n* = 3)
AUC_0-inf_, ng h/mL	27,283.3 (5087.80)	30,366.7 (6062.23)	33,614.3 (13874.13)	12,820.0 (7870.43)	32,364.4 (11900.94)	49,400.0 (8179.85)
*C*_max_, ng/mL	1986.2 (3084.77)	1119.8 (205.71)	1211.3 (293.35)	665.3 (125.13)	1213.3 (259.42)	1473.3 (47.26)
*t*_max_, h^a^	1.95 (0.92, 4.77)	2.835 (1.50, 5.00)	2.00 (0.98, 5.08)	0.82 (0.23, 0.95)	1.13 (0.95, 2.92)	2.10 (1.18, 2.17)
*t*_1/2_, h	22.20 (2.212)	23.62 (4.614)	34.87 (19.374)	36.83 (32.679)	28.07 (9.386)	21.13 (3.837)
CL, mL/h	60.15 (17.500)	75.93 (22.818)	80.90 (29.390)	148.63 (61.905)	95.82 (58.289)	65.37 (8.465)
*V*_d_, mL	1953.3 (547.53)	2091.7 (505.03)	2310.0 (514.39)	3793.3 (1866.61)	2873.3 (1530.20)	1993.3 (96.09)

^a^Median (min, max)

*AUC*_*0-inf*_ area under the concentration–time curve from zero time extrapolated to infinite time, *AUC*_*0-t*_ area under the concentration–time curve from zero time to time of last quantifiable concentration, *C*_*max*_ maximum observed concentration, *CL* total clearance, *LF* liposomal formulation, *t*_*max*_ time at which the highest drug concentration occurs, *t*_*1/2*_ terminal elimination phase half-life, *V*_*d*_ volume of distribution

#### Efficacy

**Fig. 2:**
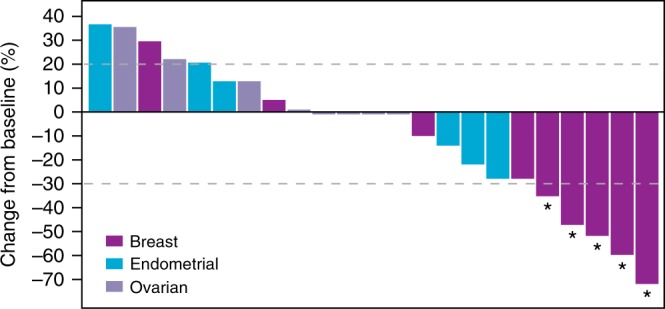
Waterfall plot displaying maximum percentage change from baseline in sums of lesion diameters in patients with breast, ovarian or endometrial cancers from the dose-expansion phase (Schedule 2). *Patients who achieved a partial response (PR)

Investigator-assessed tumour responses with eribulin-LF for schedules 1 and 2 are shown in Supplementary Table 4 and Supplementary Figure 3. Partial response was observed in 6 (10.3%) patients: 2 (10%) patients treated in Schedule 1 (1 at 1.4 mg/m^2^ and 1 at 1 mg/m^2^) and 4 (10.5%) patients treated in Schedule 2 (3 at 1.5 mg/m^2^ and 1 at 2.0 mg/m^2^). Of the 6 partial responses, 5 were in patients with breast cancer: 1 patient received Schedule 1 (1 mg/m^2^) and 4 patients received Schedule 2 (2 mg/m^2^, *n* = 1; 1.5 mg/m^2^, *n* = 3). For the 6 patients who achieved partial response, the duration of response ranged from 49 to 297 days (49, 57, 98, 125, 191 and 297 days). Four out of these six patients had ongoing response at the subsequent imaging assessment. Of note, these patients had received 1, 2 or 3 prior lines of anticancer therapy (2 patients each). Antitumour activity was observed at each dose level, and the overall objective response rate among all 58 patients for the dose-escalation and dose-expansion phases was 10.3%. Investigator-assessed tumour responses during the dose-expansion phase are shown in Fig. 2. The disease-control rate was 80% in patients with breast cancer, and 40.0% and 42.9% in patients with ovarian and endometrial cancers, respectively (Supplementary Table 4). The median duration of treatment was 8 weeks (range: 3–79 weeks) and was similar between Schedule 1 (9 weeks; range: 3–79 weeks) and Schedule 2 (8 weeks; range: 4–48 weeks).

## Discussion

In this study, the MTD for eribulin-LF was established for each schedule (Schedule 1: 1.4 mg/m^2^; Schedule 2: 1.5 mg/m^2^). Given that Schedule 2 had both a higher MTD and a higher dose intensity, which facilitates greater drug exposure and potentially increased efficacy, it was selected for the dose-expansion phase of the study.

In this study, 5 patients (8.6%) experienced any-grade hypophosphatemia (4 patients had grade 3 and 1 patient had grade 4). The 1 patient who experienced a DLT of grade 4 hypophosphatemia at cycle 1, day 8, had borderline grade 1/2 hypophosphatemia at study entry. Grade 3/4 hypophosphatemia by laboratory measurements was observed in 3.2% of patients in the phase 3 trial of the aqueous formulation of eribulin in advanced soft-tissue sarcoma.^7^ The causative mechanism of this remains unknown. Hypophosphatemia has also been observed in other recent early-phase clinical trials.^18,19^ The incidence of peripheral neuropathy (narrow and broad terms) in this study of eribulin-LF was 19%; this is lower than that reported in the two phase 3 clinical trials of eribulin in breast cancer (35% and 27.4%).^6,20^ The incidence of peripheral sensory neuropathy in this study (10.3%) was also lower than that reported in the phase 3 clinical trial of eribulin in soft-tissue sarcoma (grade 1–2, 19%; grade 3, 2%).^9^

The actual dose intensity at the MTD was slightly higher for Schedule 2 than for Schedule 1 (0.6 vs. 0.5 mg/m^2^/week per patient); however, the relative dose intensity at the MTD for Schedule 2 was lower than for Schedule 1 (0.7 vs. 1.0, respectively). This may be explained by the higher number of dose interruptions and omissions observed in Schedule 2 patients at the MTD and dose reductions due to neutropenic events. The difference between the incidence of neutropenia events in Schedule 1 and Schedule 2 (20% vs. 42.1%) was investigated, with a particular focus on 11 patients who experienced a dose interruption due to neutropenia in Schedule 2 at the eribulin-LF MTD of 1.5 mg/m^2^. Of these patients, 8 did not receive the cycle 1, day 15 dose: 7 of these omissions were due to neutropenia, and 1 was due to concurrent upper respiratory tract infection and ascites. However, the criteria for repeat dosing on day 15 included an ANC ≥ 1.5 × 10^9^/L. All 7 patients did have an ANC < 1.5 × 10^9^/L on cycle 1, day 15 but only 2 of these patients had ANC < 1.0 × 10^9^/L. In addition, all these patients proceeded with eribulin-LF dosing in cycle 2. The high number of dose interruptions in Schedule 2 for neutropenia suggests the protocol criteria for dosing on day 15 of the treatment cycle (i.e., ANC of ≥1.5 × 10^9^/L) may have been too stringent. Of the 5 patients with an ANC of < 1.5 × 10^9^/L and ANC > 1.0 × 10^9^/L, recovery from the neutropenia was rapid and allowed continued dosing, usually without the need for granulocyte-colony stimulating factor treatment, demonstrating that this dose and schedule was, in fact, tolerable. For further development, therefore, a different dose adjustment criterion for Schedule 2 could be considered. In addition, our results also suggest that if a patient cannot tolerate the 28-day schedule of eribulin-LF, a 21-day schedule may be considered.

A hypersensitivity reaction (grade 2) following constant-rate infusion occurred in the first patient enroled in the study and occurred again in the second cycle in the same patient. This event was considered a complement-activation-related pseudo allergy associated with the liposomal lipid component of the formulation.^21^ As a result, the protocol for this study was amended to introduce a stepped-escalation infusion rate for eribulin-LF, starting at an infusion rate of 0.005 mg/min for the first 10 min, which could be escalated (if tolerated without reaction) to a rate not exceeding 0.2 mg/min. Following this change in protocol, 7% of patients experienced grade 1/2 hypersensitivity reactions on a single occasion, and no grade ≥ 3 hypersensitivity reactions occurred. No prophylaxis premedication was administered to manage these events.

Eribulin-LF has a single-phase pharmacokinetic profile, which differs from the pharmacokinetic profile of the aqueous formulation of eribulin, which has been described as either biphasic^22^ or triphasic.^23^ The elimination t_1/2_ (~30 h, range: 21.1–36.8) of eribulin-LF is comparable to that of eribulin (~40−48 h), but the maximum observed concentration (*C*_max_) is approximately 5-fold greater and the AUC is approximately 40-fold greater with eribulin-LF.^22,23^ The liposomal formulation enabled longer exposure to eribulin, with the plasma concentration quantifiable up to 18 days postdose, compared with 8 days postdose for eribulin aqueous formulation.^24^ Eribulin-LF clearance (CL) and volume of distribution were much lower than those for eribulin (CL = ~3.4 L/h for eribulin and 0.08 L/h for eribulin-LF; volume of distribution of approximately 134 L for eribulin and ~2 L for eribulin-LF).^14,15^ These observations are in line with doxorubicin liposomal formulation and are the disposition characteristics of a liposomal formulation.^25^ The longer duration of exposure of eribulin-LF (18 days vs. 8 days) is also due to a ~40-fold greater AUC following the same magnitude of eribulin dose.

Several liposomal formulations of anticancer drugs have been approved in various tumour types, including a PEGylated liposomal formulation of doxorubicin.^26^ The liposomal formulation of doxorubicin demonstrated a lower volume of distribution (4.1 vs. 254 L) and clearance (0.1 vs. 45.3 L/h), and a drug concentration in the tumour that was 4–16 times higher than free doxorubicin.^25^ Moreover, cardiotoxicity was reduced with the PEGylated liposomal formulation.^26,27^ Given the potential for reduced toxicity with liposomal formulations, eribulin-LF may facilitate combinations with other anticancer agents.

Overall, no new or unexpected safety signals were identified with eribulin-LF compared to the eribulin aqueous formulation. The objective response rate in patients with breast cancer in this study compared favourably with data from pooled analyses from 2 phase 3 studies of eribulin.^10^ Although these results are encouraging, the sample size of the breast cancer group was small and, therefore, robust conclusions could not be drawn. Further investigation of eribulin-LF in breast cancer as a single-agent and in combination with other therapeutics is under consideration.

## Conclusions

This first-in-human study met its primary objective of establishing the MTD of eribulin-LF at 1.4 mg/m^2^ for Schedule 1 (day 1 every 21 days) and 1.5 mg/m^2^ for Schedule 2 (day 1 and day 15 every 28 days). In this study, eribulin-LF was well tolerated with an AE profile similar to that of aqueous eribulin. Safety results were similar across the tumour types evaluated. The pharmacokinetic profile of eribulin-LF showed the anticipated characteristics of a liposomal formulation. Eribulin-LF showed preliminary activity in several tumour types, including breast, endometrial and ovarian cancers.

## Supplementary information

Supplementary Material

Supplementary Figure 1

Supplementary Figure 2

Supplementary Figure 3
