# Supplementary material for: Phase 1 dose-finding and pharmacokinetic study of eribulin-liposomal formulation in patients with solid tumours
Source: Br J Cancer. 2019 Jan 25;120(4):379–86. doi: 10.1038/s41416-019-0377-x (PMC6461749; doi:10.1038/s41416-019-0377-x)
Supplement: Supplementary file 1 — Supplementary Material [file 41416_2019_377_MOESM1_ESM.docx]

**Supplementary information**

Supplementary .pdf file contains a list of the criteria for dosing and dose modifications. A series of Supplementary Tables showing dose modification, summary of AEs, and best overall responses. Figures include a CONSORT diagram, duration of treatment, and changes in tumor size.

*Criteria for dosing and dose modifications*

Dose modifications or interruptions were conducted at the occurrence of any of the following events:

- Grade 4 neutropenia (ANC <1.0 x 10^9^/L [1000/mm^3^]) >5 days; grade 3 or 4 febrile neutropenia and/or infection requiring treatment with antibiotics and/or growth factors; grade 4 thrombocytopenia (platelet count <25 x 10^9^/L [25 000/mm^3^]); grade 3 thrombocytopenia requiring platelet or blood transfusion or both.
- Grade 3 or 4 nonhematologic toxicities that recovered to grade ≤2 (except for inadequately treated nausea and/or vomiting) within 7 days, with or without maximal supportive care.
- If recovery was observed prior to the next scheduled dose, eribulin-LF dosing was restarted with a dose reduction.
- In case recovery had not occurred prior to the next scheduled dose, the next administration of eribulin-LF was to be omitted and eribulin-LF dosing was resumed at the next scheduled dose date with a dose reduction.
- However, if the above grade 3 or 4 hematologic toxicities did not recover to grade ≤ 2 within 14 days, the patient was to discontinue treatment. In case the patient was deemed to have clinical benefit, eribulin-LF treatment could be continued; however, each case must have been discussed with the sponsor.

Dose reductions are summarized in **Supplementary Table 1**.

**Supplementary Table 1.** Eribulin-LF dose modification for hematologic toxicities and/or grade 3/4 nonhematologic toxicities

| **Eribulin-LF Starting Dose** | **1^st^ Event** | **2^nd^ Event** | **3^rd^ Event** |
| --- | --- | --- | --- |
|  | **Dose Adjustment** | | |
| 1.0 mg/m^2^ | Reduce to 0.7 mg/m^2^ | **Discontinue** | -- |
| 1.5 mg/m^2^ | Reduce to 1.1 mg/m^2^ | Reduce to 0.7 mg/m^2^ | **Discontinue** |
| 2.0 mg/m^2^ | Reduce to 1.5 mg/m^2^ | Reduce to 1.1 mg/m^2^ | Reduce to 0.7 mg/m^2^ |
| 2.5 mg/m^2^ | Reduce to 1.9 mg/m^2^ | Reduce to 1.4 mg/m^2^ | Reduce to 1.1 mg/m^2^ |
| 3.0 mg/m^2^ | Reduce to 2.3 mg/m^2^ | Reduce to 1.7 mg/m^2^ | Reduce to 1.3 mg/m^2^ |
| 3.5 mg/m^2^ | Reduce to 2.6 mg/m^2^ | Reduce to 1.9 mg/m^2^ | Reduce to 1.4 mg/m^2^ |

**Supplementary Table 2**. Overview of TEAEs in Schedule 1 and Schedule 2

| **TEAE n, (%)** | **Schedule 1 (n = 20)** | **Schedule 2 (n = 38)** |
| --- | --- | --- |
| Patients with TEAEs | 20 (100.0) | 38 (100.0) |
| Patients with treatment-related TEAEs | 18 (90.0) | 34 (89.5) |
| Patients with serious TEAEs | 9 (45.0) | 15 (39.5) |
| Patients with grade 3 or 4 TEAE | 12 (60.0) | 16 (42.1) |
| Patients with grade 5 (fatal) TEAE | 0 | 0 |
| Patients with TEAEs leading to drug-dose adjustment | 8 (40.0) | 24 (63.2) |
| Patients with TEAEs leading to drug withdrawal^a^ | 3 (15.0) | 2 (5.3) |
| Patients with TEAEs leading to dose reduction | 1 (5.0) | 3 (7.9) |
| Patients with TEAEs leading to dose interruption | 5 (25.0) | 23 (60.5) |

^a^Schedule 1 (abdominal distension, ascites, pyrexia, blood bilirubin levels increased, decreased appetite, dyspnea, and deep vein thrombosis); Schedule 2 (abdominal distension, fatigue, and vaginal hemorrhage).

TEAE, treatment-emergent adverse event.

**Supplementary Table 3**. Treatment-related SAEs

| **Patients With SAE, n (%)** | **Schedule 1 (n = 20)** | **Schedule 2 (n = 38)** | **All patients (n = 58)** |
| --- | --- | --- | --- |
| Pyrexia | 2 (10.0) | 0 | 2 (3.4) |
| Neutropenia^a^ | 1 (5.0) | 3 (7.9)^a^ | 4 (6.9) |
| Febrile neutropenia^a^ | 1 (5.0) | 2 (5.3) | 3 (5.2) |
| Neutropenic sepsis | 1 (5.0) | 1 (2.6) | 2 (3.4) |
| Drug hypersensitivity | 1 (5.0) | 0 | 1 (1.7) |
| Hypophosphatemia^a^ | 1 (5.0) | 0 | 1 (1.7) |
| Transaminase levels increased^a^ | 1 (5.0) | 0 | 1 (1.7) |
| Alanine aminotransferase levels increased^a^ | 0 | 1 (2.6) | 1 (1.7) |
| Anemia | 0 | 1 (2.6) | 1 (1.7) |
| Cholecystitis acute | 0 | 1 (2.6) | 1 (1.7) |
| Fatigue | 0 | 1 (2.6) | 1 (1.7) |
| Malaise | 0 | 1 (2.6) | 1 (1.7) |
| Stomatitis^a^ | 0 | 1 (2.6) | 1 (1.7) |
| Upper respiratory tract infection | 0 | 1 (2.6) | 1 (1.7) |

^a^SAE that was identified as a dose-limiting toxicity in the dose-escalation phase of the study.

SAE, serious adverse event.

**Supplementary Table 4**. Best overall response to eribulin-LF by investigator assessment based on RECIST version 1.1

| **Best Overall Response, n (%)** | **Schedule 1 (n = 20)** | **Schedule 2 (n = 38)** | **Breast (n = 10)** | **Ovarian (n = 10)** | **Endometrial (n = 7)** |
| --- | --- | --- | --- | --- | --- |
| CR | 0 | 0 | 0 | 0 | 0 |
| PR | 2 (10.0) | 4 (10.5) | 5 (50.0) | 0 | 0 |
| SD | 10 (50.0) | 10 (26.3) | 3 (30.0) | 4 (40.0) | 3 (42.9) |
| PD | 7 (35.0) | 19 (50) | 2 (20.0) | 5 (50.0) | 3 (42.9) |
| Unknown | 1 (5.0) | 5 (13.2) | 0 | 1 (10.0) | 1 (14.3) |
| ORR (CR + PR)  95% CI | 2 (10.0)  (1.2, 31.7) | 4 (10.5)  (2.9, 24.8) | 5 (50.0)  -- | 0  -- | 0  -- |
| DCR (CR + PR + SD)  95% CI | 12 (60)  (36.1, 80.9) | 14 (36.8)  (21.8, 54.0) | 8 (80.0)  -- | 4 (40.0)  -- | 3 (42.9)  -- |
| CBR (CR + PR + durable SD^a^)  95% CI | 4 (20.0)  (5.7, 43.7) | 5 (13.2)  (4.4, 28.1) | --  -- | --  -- | --  -- |

^a^SD ≥ 6 months.

The tumor assessment is based on RECIST version 1.1.

CBR, clinical benefit rate; CI, confidence interval; CR, complete response; DCR, disease-control rate; ORR, objective response rate; PD, progressive disease; PR, partial response; RECIST, Response Evaluation Criteria In Solid Tumors; SD, stable disease.

**Supplementary Figure 1**. CONSORT diagram


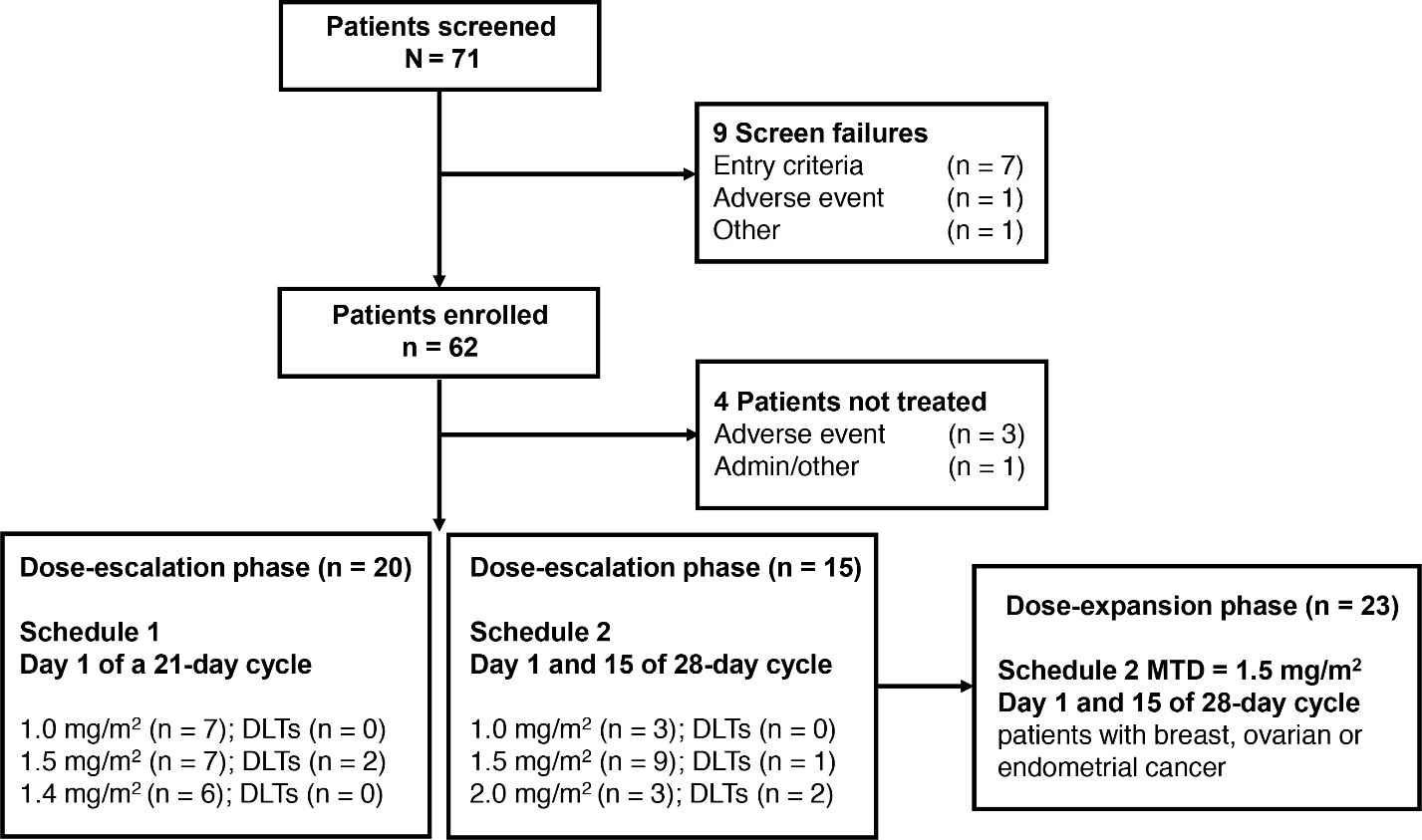


CONSORT, Consolidated Standards of Reporting Trials; DLT, dose-limiting toxicity; MTD, maximum tolerated dose.

**Supplementary Figure 2**. Duration of treatment


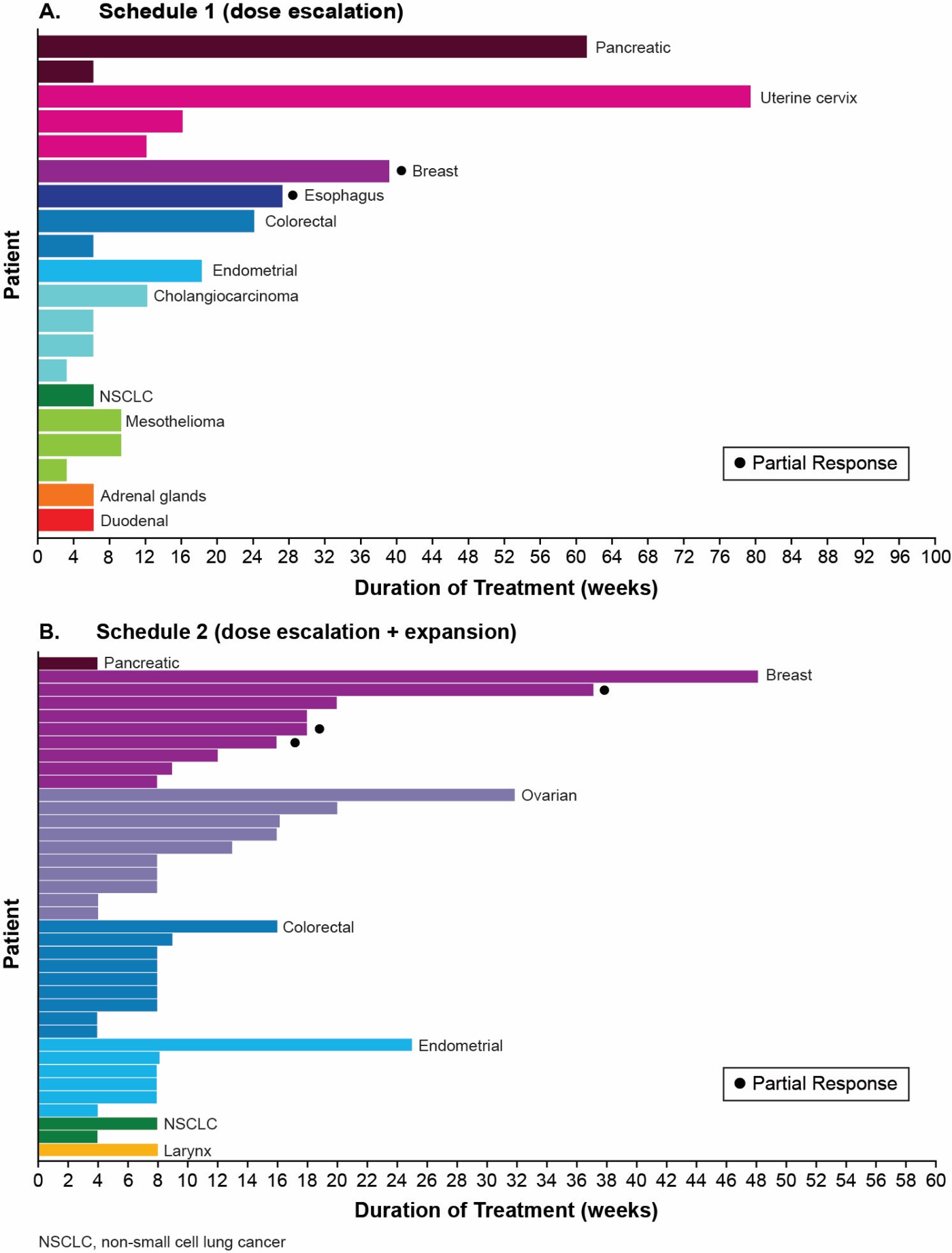


**Supplementary Figure 3.** Waterfall plot of maximum percentage change from baseline in tumor size for Schedule 1 (dose-escalation phase) and Schedule 2 (dose-escalation and dose-expansion phases)—safety analysis set. *Numbers above bars indicate eribulin-LF dose in mg/m^2^

^
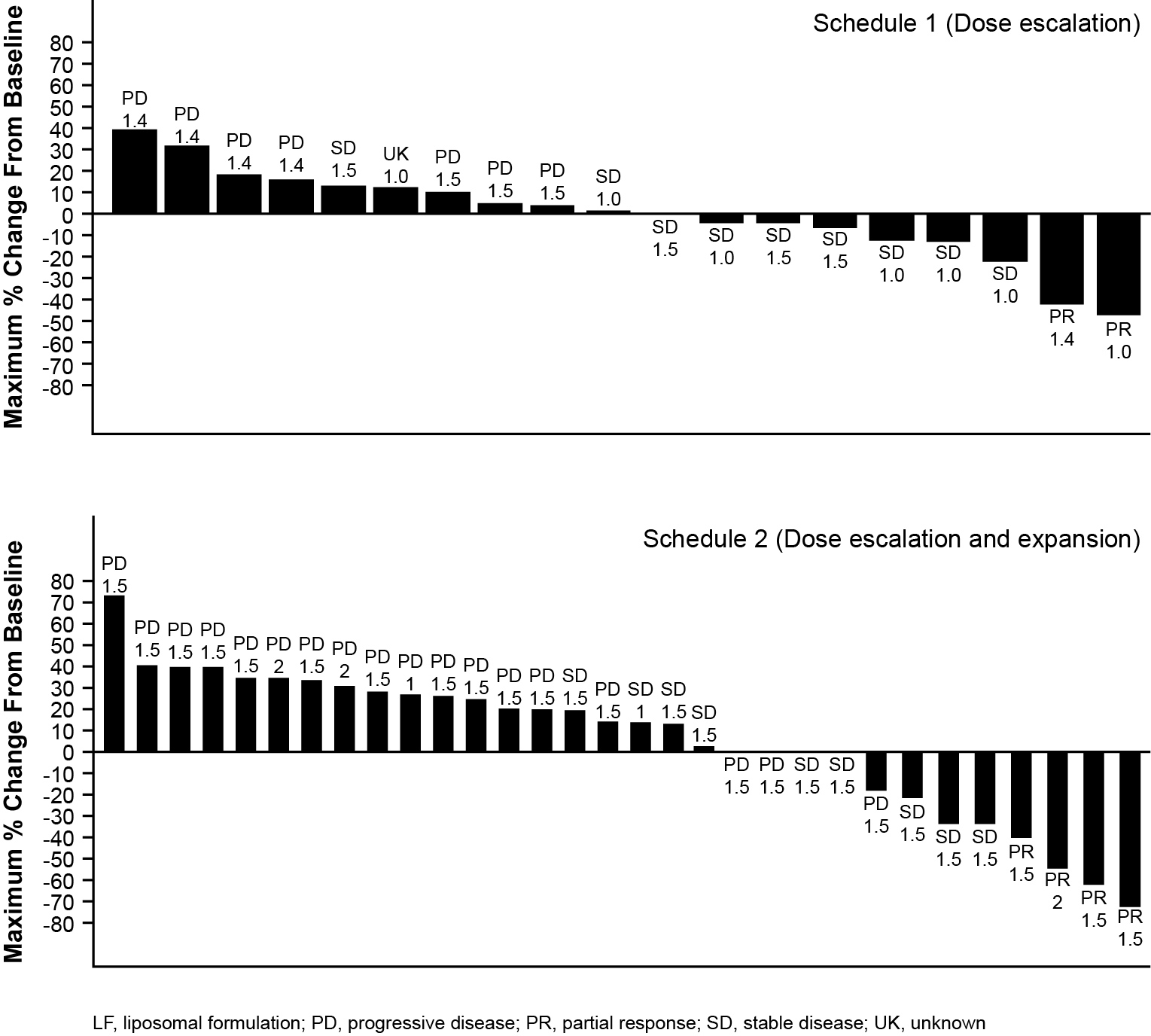
^
