# Supplementary figures and images for: Phase 1 dose-finding and pharmacokinetic study of eribulin-liposomal formulation in patients with solid tumours
Source: Br J Cancer. 2019 Jan 25;120(4):379–86. doi: 10.1038/s41416-019-0377-x (PMC6461749; doi:10.1038/s41416-019-0377-x)

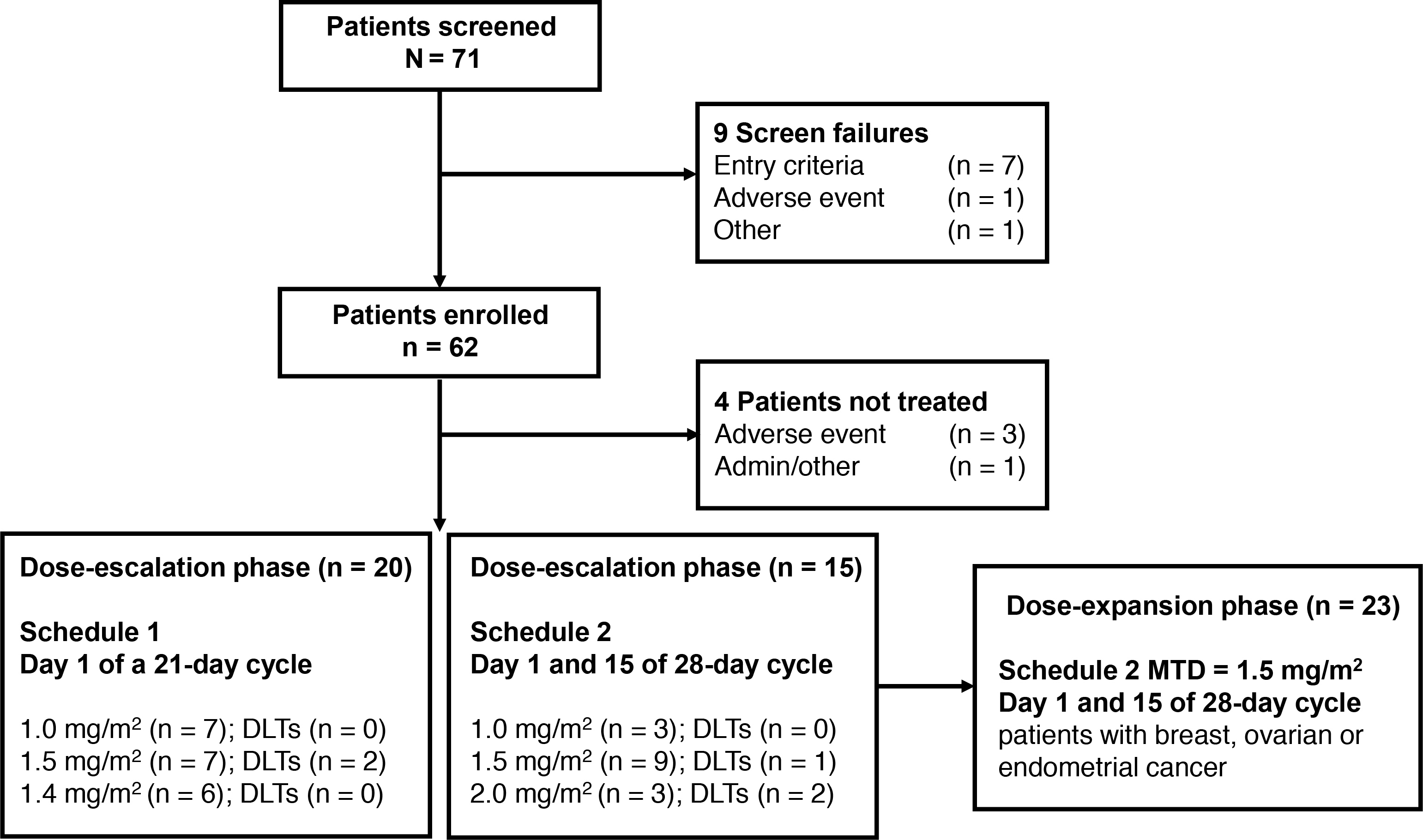

Supplement: Supplementary file 2 — Supplementary Figure 1 [file 41416_2019_377_MOESM2_ESM.jpg]

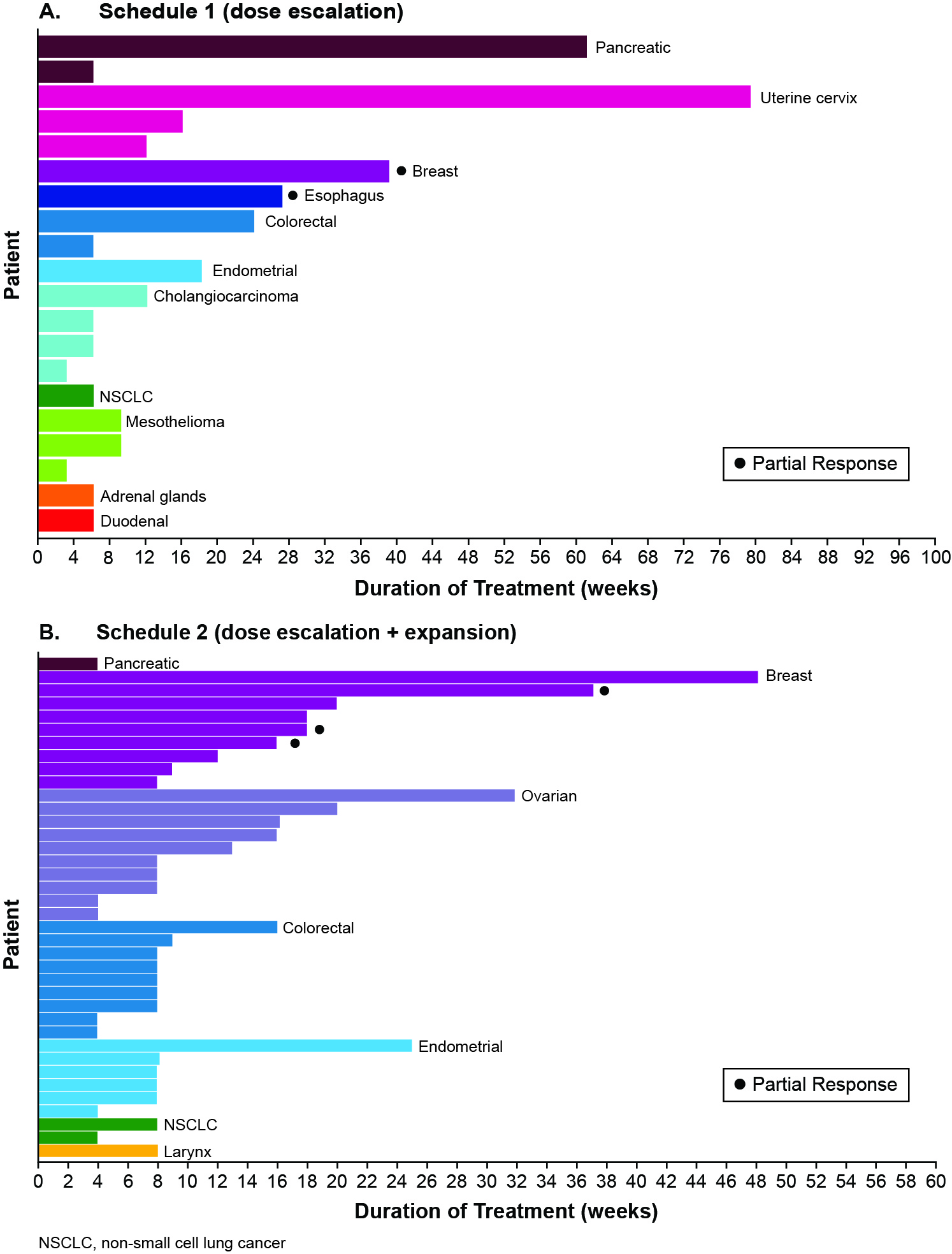

Supplement: Supplementary file 3 — Supplementary Figure 2 [file 41416_2019_377_MOESM3_ESM.jpg]

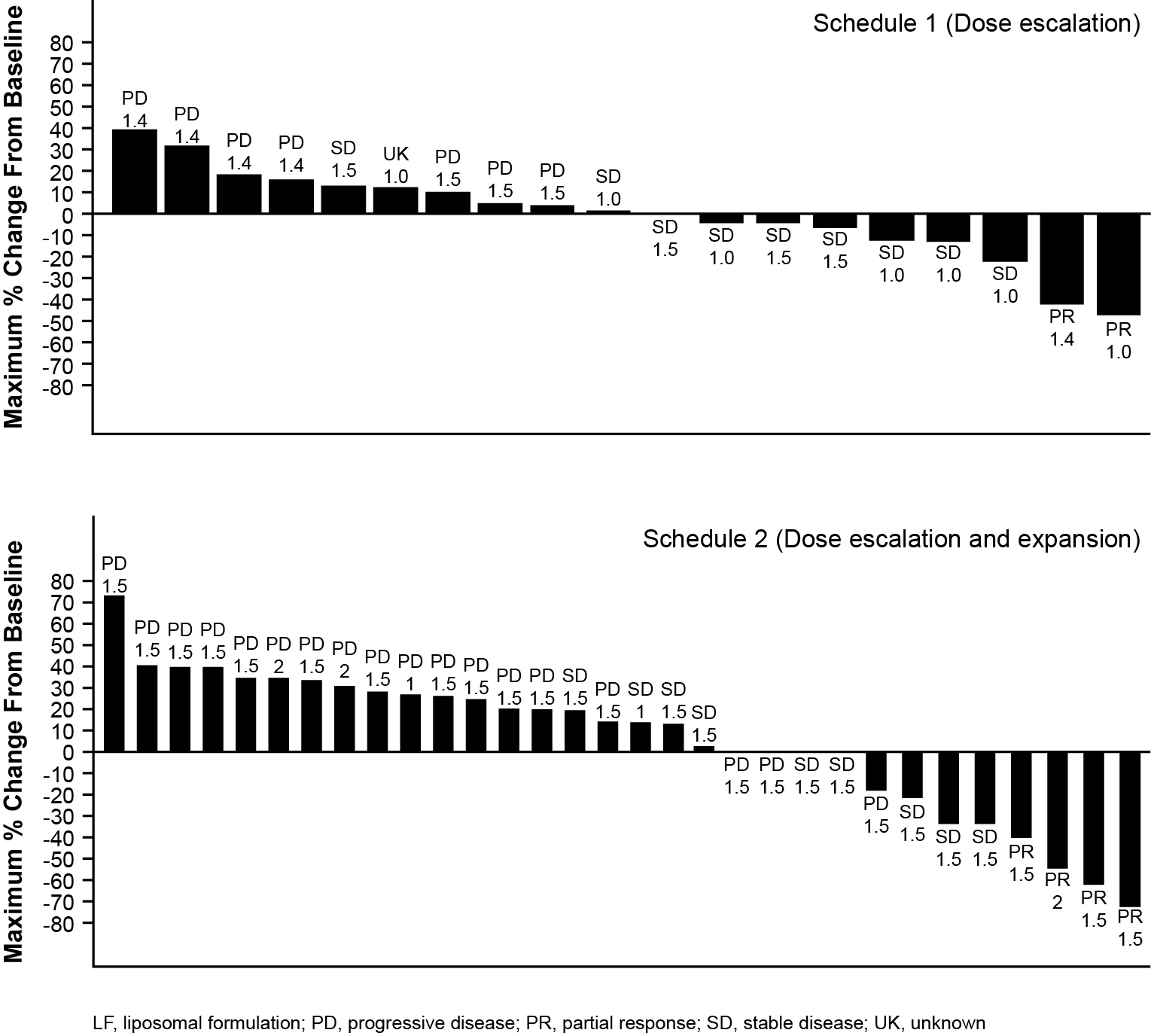

Supplement: Supplementary file 4 — Supplementary Figure 3 [file 41416_2019_377_MOESM4_ESM.jpg]
